# Effect of hyaluronic acid-enriched transfer medium on frozen–thawed embryo transfer outcomes in RIF patients: a single-centre retrospective study

**DOI:** 10.3389/fendo.2023.1170727

**Published:** 2023-07-03

**Authors:** Qiang Yan, Mei Zhao, Fan Hao, Ruru Zhao, Xiaoming Teng, Bin He, Chong Zhu, Zhiqin Chen, Kunming Li

**Affiliations:** ^1^ Reproductive Medicine Center, Shanghai First Maternity and Infant Hospital, School of Medicine, Tongji University, Shanghai, China; ^2^ Shanghai Key Laboratory of Maternal Fetal Medicine, Shanghai First Maternity and Infant Hospital, School of Medicine, Tongji University, Shanghai, China; ^3^ Shanghai Institute of Maternal-Fetal Medicine and Gynecologic Oncology, Shanghai First Maternity and Infant Hospital, School of Medicine, Tongji University, Shanghai, China

**Keywords:** hyaluronic acid, recurrent implantation failure, clinical pregnancy rate, live birth rate, frozen embryo transfer

## Abstract

**Introduction:**

Many patients who undergo assisted reproductive technology (ART) suffer from recurrent implantation failure (RIF). The addition of hyaluronic acid (HA) to the transfer medium is one of several methods to improve pregnancy outcomes. We investigated whether HA could improve the live birth and clinical pregnancy rates of RIF patients.

**Methods:**

This study included 248 RIF patients, who were divided into two groups: the control (CTL) group (n=137), which received transfer medium without HA, and the HA group (n=111), which received transfer medium with HA. The two groups were compared according to the ART outcome.

**Results:**

The primary outcomes were the clinical pregnancy and live birth rates. Secondary outcomes include a positive urine pregnancy test, the implantation, ongoing pregnancy, multiple pregnancy, clinical miscarriage, and ectopic pregnancy rates, foetal or congenital defects, obstetric complications, infant birth weight and any related adverse events. Regarding the primary outcomes, the clinical pregnancy rate was significantly higher in the HA group than in the control group, and there was no significant difference in the live birth rate (LBR) between the HA and control groups. Regarding the secondary outcomes, the implantation, multiple pregnancy and ectopic pregnancy rates were similar between the two groups.

**Discussion:**

Our findings supported the conclusion that HA can improve the clinical pregnancy rate of patients with RIF undergoing FET cycles, but the live birth rate was not significantly improved with the addition of HA to the traditional transfer medium.

## 1 Introduction

Since the birth of the world’s first infant conceived by *in vitro* fertilisation-embryo transfer (IVF-ET) in 1978, assisted reproductive technology (ART) has undergone remarkable development (1). Despite the rapid development, the current pregnancy rate is still maintained at a low level of approximately 40%–50% (2). Among IVF patients, 10% suffer from recurrent implantation failure (RIF), which places physical and financial pressure on the patients (3, 4). Thus, improving the clinical outcomes of patients, especially those with RIF, is still an important issue.

Successful embryo implantation requires mutual recognition and interaction between the endometrium and the embryo (4, 5). Thus, many studies have proposed methods related to these two aspects (6): preimplantation genetic testing (PGT), endometrial receptivity assay (ERA), and so on. In addition, previous studies have demonstrated that some components in the transfer medium of the embryos affect the pregnancy outcomes of patients undergoing ART (7). Some researchers have suggested polyvinylpyrrolidone (8), polyvinylalcohol (9), and HA (10, 11) as available options. In the past few decades, only hyaluronic acid has been used as an additive to transplantation fluid in embryo laboratories (12, 13). However, the efficacy of HA is still controversial.

HA is an abundant glycosaminoglycan that exists in the female reproductive system, including the fallopian tubes, follicles, and endometrium (14–16). The CD44 receptor of HA is expressed on the surface of the embryo and the endometrium (17). Many studies have also shown that HA can improve endometrial receptivity and induce embryo implantation in animals (18, 19). Therefore, in ART, HA is added to the transfer medium to support embryo implantation. EmbryoGlue (Vitrolife, Denver, CO, USA), which contains various substances with the active ingredient hyaluronan, was developed and is considered to improve IVF outcomes by some researchers (20). Many reproductive centres around the world have also conducted studies on whether HA can improve the implantation rate (IR) and pregnancy rate (PR); however, there have been no consistent conclusions (21).

Improving the pregnancy outcome of patients with recurrent implantation failure is an urgent problem that needs to be solved (22). At present, the diagnostic criteria for RIF are not uniform (23). The more generally recognised criteria of RIF are as follows: age less than 40 years and three transplantation cycles (including fresh embryo transfer and frozen–thawed embryo transfer) with more than four high-quality embryos transferred without pregnancy (6, 22, 24). Most of the HA studies were conducted in fresh transfer cycles, and few studies focused on the application of HA in frozen–thawed embryo transfer (FET) cycles, especially in patients with RIF (25). The effect of HA-enriched transfer medium may be different in FET cycles and fresh cycles. Embryo endometrium asynchrony is present in fresh cycles because of supraphysiologic levels of oestradiol and progesterone. One randomised controlled trial (RCT) compared the effect of hyaluronic acid (HA)-enriched transfer medium versus standard medium on the live birth rate of FET cycles and showed no significant difference in the clinical pregnancy rate or live birth rate between the two groups of patients from the general population undergoing IVF (13). However, this study did not include women with recurrent implantation failure, who might have benefited from the use of HA-enriched medium.

Therefore, we performed a retrospective study to examine the effect of HA-enriched transfer medium on the clinical outcomes of FET cycles in patients with RIF. We hypothesised that the use of an HA-enriched transfer medium would significantly increase the clinical pregnancy and live birth rates in patients undergoing FET cycles.

## 2 Methods

### 2.1 Patients

We analysed data from infertile women who underwent IVF/intracytoplasmic sperm injection (ICSI) cycles at the Center of Assisted Reproduction, Shanghai First Maternity and Infant Health Hospital, Tong Ji University, between April 2017 and April 2020. Women were included if they met the following inclusion criteria: i) were younger than 40 years, ii) had failed to achieve a clinical pregnancy after the transfer of at least four good-quality embryos in a minimum of three fresh or frozen cycles, iii) had an endometrial thickness ≥8 mm on the day of FET, and iv) had a normal uterine cavity shown on hysterosalpingogram or hysteroscopy. Women were excluded if they had i) used donor eggs/sperm, ii) had hydrosalpinges shown on scanning that had not been treated, iii) had moderate or severe endometriosis, iv) had an abnormal chromosome (or their partner had an abnormal chromosome), v) had a congenital uterine anomaly, or vi) had unclear information on previous transfer cycles.

All patients were undergoing frozen–thawed embryo transfer and received either HA-enriched (HA group) or conventional transfer medium (CTL group) at the discretion of the attending physicians or based on the wishes of the couple after extensive counselling.

### 2.2 Ovarian stimulation and IVF

Most patients started IVF with ovarian stimulation using the long/short agonist or antagonist protocols, progestin-primed ovarian stimulation (PPOS), or mild stimulation. For the long protocol, gonadotropin-releasing hormone analogue (GnRHa) was given for pituitary desensitisation. On Days 2–3 of their menstrual cycles, the patients underwent transvaginal ultrasound examination and serum oestradiol measurement. Human menopausal gonadotropin (hMG) (Lebaode, Lizhu, China) or recombinant follicle-stimulating hormone (FSH) (Puregon, Organon, Dublin, Ireland, or Gonal F, Merck Serono S.p.A., Modugno, Italy) was given at 150–225 IU per day based on the antral follicle count, maternal age, and previous ovarian response, according to the standard operating procedures of the centre. For PPOS, medroxyprogesterone (MPA; 10 mg/day; Shanghai Xinyi Pharmaceutical Co., Shanghai, China) was also given afterwards on the same day. The ovarian response was monitored by serial transvaginal scanning with or without hormone monitoring. Further dosage adjustments were based on the ovarian response at the discretion of the clinicians in charge. For the antagonist protocol, 0.25 mg of antagonist daily (Orgalutran, Organon, Dublin, Ireland) was given from the sixth day of ovarian stimulation until the day of ovulation trigger. Mild stimulation was used in poor responders, and clomifene citrate 100 mg was given for 5 days followed by hMG 150 IU per day until the day of ovulation trigger.

When three leading follicles reached ≥18 mm in diameter, triptorelin (0.1 mg; Decapeptyl, Ferring Pharmaceuticals, Netherlands) and human chorionic gonadotropin (hCG) (2,000 IU; Lizhu Pharmaceutical Trading Co., Zhuhai, China) or Ovidrel 250 μg (Merck Serono S.p.A., Modugno, Italy) were given to trigger the final maturation of oocytes. Oocyte retrieval was performed approximately 36 h later.

### 2.3 Fertilisation and embryo evaluation

Approximately 2 h after oocyte retrieval, each oocyte was inseminated with approximately 20,000–30,000 motile spermatozoa. If the total number of motile sperm was <10^5^ after washing or <1% of sperm had normal morphology, ICSI was performed. Oocytes were decoronated and checked for the presence of two pronuclei to confirm fertilisation. Embryos were graded on Day 3 after retrieval as grade 1 to grade 6 according to the evenness of each blastomere and the percentage of fragmentation (26). Embryos with four cells (for Day 2 embryos) or eight cells (for Day 3 embryos) and of grade 1 or 2 were regarded as “top quality embryos” in this study (13). Some non-top-quality embryos were placed in extended culture until they reached the blastocyst stage. Blastocysts were graded using the scoring system described by Gardner (27). Expanded, hatching, or hatched blastocysts (expansion grade 4 or above) with an inner cell mass and a trophectoderm grade of AA, AB, or BA were regarded as “top-quality blastocysts”.

Embryo transfer was performed on Day 3 or 5 after oocyte retrieval, and good-quality surplus embryos (grades 1 to 4) or blastocysts (expansion stage 3 or above, with either an inner cell mass or a trophectoderm score of B or above) were cryopreserved. For women at risk for ovarian hyperstimulation syndrome, fresh embryo transfer was cancelled, and all embryos were cryopreserved.

### 2.4 Cryopreservation and frozen–thawed embryo transfer

Surplus embryos of grades 1 to 4 were cryopreserved using a vitrification protocol on the day of embryo transfer. Patients who did not become pregnant in the stimulated IVF cycle and those who postponed embryo transfer underwent FET at least 2 months after the stimulated cycle if they had at least one frozen embryo. FETs were carried out in natural cycles for ovulatory women and in clomiphene/letrozole-induced cycles or hormone replacement cycles for anovulatory women.

#### 2.4.1 Natural cycles

Women with regular cycles had daily blood tests to identify the day of the luteinising hormone (LH) surge as described previously (28), which was defined as the elevation of the LH level to two times the average level of the previous 3 days, and the absolute level of the LH surge was greater than 20 IU/L. Transvaginal ultrasonography was performed to measure the endometrial thickness 1 day after the LH surge. If the endometrial thickness reached 8 mm or more, luteal support was initiated.

#### 2.4.2 Hormonal cycles

Women received oral oestradiol 4–6 mg daily for 12–14 days for endometrial priming, followed by transvaginal ultrasonography to evaluate endometrial thickness. If the endometrial thickness was ≥8 mm, vaginal micronised progesterone 100 mg three times daily was initiated. If the woman became pregnant, oral oestradiol and vaginal progesterone were continued after FET until 12 weeks of gestation.

#### 2.4.3 Letrozole/clomiphene cycles

Some women with irregular menstrual cycles received clomiphene 50 mg or letrozole 2.5 mg daily for 5 days. Depending on the growth of the follicles, HMG was injected to promote follicle growth. When the follicle developed to 16–20 mm in diameter with a standard endometrium thickness (≥8 mm), hCG was used to induce ovulation.

Vitrification was performed with MediCult Vitrification Cooling (Origio, Måløv, Denmark) using ethylene glycol, propylene glycol, and sucrose as cryoprotectants. Embryos were vitrified one by one at room temperature. For the warming procedure following vitrification, one by one, the straw was cut, and the capillary was pulled from the straw out of the liquid nitrogen and immediately warmed using MediCult Vitrification Warming (Origio, Måløv, Denmark). After warming, the embryos were transferred to a culture dish for evaluation and further development. Only embryos with more than 50% of blastomeres present after thawing were transferred in FET cycles. Again, up to two embryos or blastocysts were transferred in each FET cycle.

### 2.5 FET and EmbryoGlue^®^ treatment of embryos

For women in the HA group, EmbryoGlue (Vitrolife, Gothenburg, Sweden) was used as the embryo transfer medium, while for those in the control group, G-2 (Vitrolife) medium supplemented with HSA solution (Vitrolife) was used. This supplemented G-2 medium is normally used in our laboratory and served as a control, while EmbryoGlue is an HA-enriched embryo transfer medium that was developed from G-2. The main difference between the two media is that EmbryoGlue contains a higher concentration of HA (0.5 *vs.* 0.125 mg/ml). Furthermore, EmbryoGlue contains 2.5 mg/ml of recombinant human serum albumin (HSA), while the control medium contains 10 mg/ml of HSA (28).

On the morning of FET, frozen embryos or blastocysts were thawed or warmed and incubated for at least 10 min in the transfer medium according to the group assignment before being transferred in the same medium.

### 2.6 Follow-up

A urine pregnancy test was performed 14 days after embryo transfer. If the pregnancy test was positive, transvaginal ultrasonography was performed 2 and 4 weeks later to locate the pregnancy and check foetal viability. Patients were referred for antenatal care for an ongoing pregnancy at 8 weeks.

The obstetric outcomes were traced from the electronic patient record system of the patients delivered in public hospitals. The outcome of the pregnancy, the number of babies born, birth weight, gestational age at delivery, and obstetric complications were recorded.

### 2.7 Study outcomes

The primary outcomes were clinical pregnancy (the presence of an intrauterine gestational sac at 6 weeks of gestation on ultrasonography) and a live birth beyond 22 weeks of gestation (29). Secondary outcomes included a positive urine pregnancy test, ongoing pregnancy (a viable pregnancy beyond 8 weeks gestation), multiple pregnancies, clinical miscarriage, ectopic pregnancy, miscarriage rates, foetal or congenital defects, obstetric complications, infant birth weight, and any related adverse events. The implantation rate was calculated as the total number of gestational sacs divided by the total number of embryos transferred.

### 2.8 Statistical analysis

Continuous variables are given as mean ± SD if normally distributed and as median + interquartile range if not normally distributed. Statistical comparison was carried out by Student’s t-test; the Mann−Whitney U test was used for continuous variables, and the chi-square test was used for categorical variables, where appropriate. Statistical analyses were performed using the Statistical Package for the Social Sciences (SPSS Inc., Version 24.0, Chicago, IL, USA). A two-tailed value of *p* < 0.05 was considered statistically significant.

## 3 Results

### 3.1 Demographic and cycle characteristics

A total of 248 women with RIF were enrolled in this study from April 2017 to April 2020; 111 women were included in the HA group, and 137 women were included in the control group. As shown in **Table 1**, the two groups were comparable with respect to maternal age at the time of IVF, the proportion of primary infertility, the duration and cause of infertility, the antral follicle count, the ovarian stimulation protocol and the total gonadotropin dose, serum oestradiol and progesterone levels on the day of trigger, the number of oocytes collected, the number of oocytes fertilised, and the number of embryos frozen. However, the body mass index (BMI) of the women in the control group was significantly higher than that of the women in the HA group (21.53 ± 2.34 *vs.* 20.81 ± 2.32, *p* = 0.047). In the FET cycles, no significant differences between the two groups were found in the endometrial preparation, serum oestradiol level, endometrial thickness, stage of embryo replacement, number of embryos replaced, or proportion of women with top-quality embryos/blastocysts transferred (*p* > 0.05, **Table 2**).

**Table 1:** Patient characteristics in FET cycles.

The index stimulated IVF cycles	HA group (n = 111)	Control group (n = 137)	*p*
Age at IVF (years)	34.41 ± 4.42	33.66 ± 4.42	0.168
Primary infertility	57 (51.4)	68 (49.6)	0.788
Cause of infertility, n (%)			0.306
Tubal Anovulatory Male Endometriosis Unexplained	85 (76.6) 5 (4.5) 14 (12.6) 5 (4.5) 2 (1.8)	99 (72.2) 14 (10.2) 11 (8.0) 9 (6.6) 4 (2.9)	
Duration of infertility (years)	2 (1–4)	2 (1–4)	0.106
Body mass index (kg/m^2^)	20.81 ± 2.32	21.53 ± 2.34	0.047
Previous transfer cycles n (%)			0.571
3	73 (65.7)	95 (69.3)	
4	20 (18)	27 (19.7)	
5	11 (9.9)	10 (7.3)	
≥6	7 (6.3)	5 (3.6)	
Number of previous embryos transferred	5 (4–6)	5 (4–6)	0.553
Proportion of women who underwent ICSI	64 (57.7)	66 (48.2)	0.137
Antral follicle count	16 (12–20)	15 (11–20)	0.087
Stimulation regimen			0.663
Long GnRH agonist	28 (25.0)	41 (29.9)	
GnRH antagonist	45 (40.5)	61 (44.5)	
Mild stimulation	21 (18.8)	22 (15.3)	
PPOS	15 (13.4)	11 (8.8)	
Short agonist	2 (1.8)	2 (1.4)	
Duration of FSH stimulation (days)	9 (8–10)	9 (8.25–10.75)	0.298
Total dosage of FSH used (IU)	1,800 (1,268.75–2,268.75)	1,800 (1,350–2,250)	0.551
Serum oestradiol level on the day of trigger (pmol/L)	2,288.06 (1,652.75–3,575.69)	2,625.50 (1,583.52–3,572.75)	0.895
Serum progesterone level on the day of trigger (nmol/L)	0.9 (0.63–1.25)	0.9 (0.70–1.22)	0.735
Number of oocytes collected	12 (7–15)	11 (6.25–17)	0.701
Number of oocytes fertilised	9 (5–12)	9 (5–12.75)	0.925
Number of embryos frozen	4 (2–6)	4 (2–6)	0.571
Number of fresh embryo transfers performed	98 (88.3)	100 (73)	0.003

Data are presented as the number (percentage) or mean ± SD.

FET, frozen–thawed embryo transfer; HA, hyaluronic acid; IVF, *in vitro* fertilisation; ICSI, intracytoplasmic sperm injection; FSH, follicle-stimulating hormone; GnRH, gonadotropin-releasing hormone; PPOS, progestin-primed ovarian stimulation.

**Table 2:** Characteristics of the FET cycles.

	HA group (n = 111)	Control group (n = 137)	*p*-Value
Endometrial preparation			0.317
Natural cycle Hormonal cycle Mild stimulation	18 (16.2) 90 (81.1) 3 (2.7)	16 (11.7) 113 (82.5) 8 (5.8)	
Peak serum oestradiol level (pmol/L)	249.54 (198–673)	261 (188–479)	0.459
Endometrial thickness (mm)	9 (8–10)	9 (8–10)	0.433
Stage of embryo replacement			0.816
Cleavage stage Blastocyst	64 (57.7) 47 (42.3)	81 (59.1) 56 (40.9)	
Number of embryos/blastocysts transferred			0.261
One Two	40 (36) 71 (64)	59 (43.1) 78 (56.9)	
Women with top-quality embryos/blastocysts transferred	73 (65.7)	87 (63.5)	0.711

Data are presented as the number (percentage) or mean (data range).

FET, frozen–thawed embryo transfer; HA, hyaluronic acid.

### 3.2 Primary outcome

The clinical pregnancy rates were significantly higher in the HA group than in the control group (39.6% *vs.* 29.7%; *p* = 0.048; relative risk 1.711; 95% CI 1.004–2.917). There was no significant difference in the live birth rate (LBR) between the HA and control groups (34.2% *vs.* 25.5%; *p* = 0.136; relative risk 1.517; 95% CI 0.876–2.626) (**Table 3**).

**Table 3:** Comparison of pregnancy outcomes in women undergoing FET cycles.

	HA group (n = 111)	Control group (n = 137)	*p*-Value	Relative risk (95% CI)
Positive pregnancy test	47 (42.3)	48 (35)	0.239	1.362 (0.814–2.278)
Clinical pregnancy rate	44 (39.6)	38 (27.7)	0.048	1.711 (1.004–2.917)
Live birth rate	38 (34.2)	35 (25.5)	0.136	1.517 (0.876–2.626)
Clinical miscarriage rate	12.8 (6/47)	6.3 (3/48)	0.278	2.195 (0.515–9.349)
Multiple pregnancy rate	11.4 (5/44)	21.1 (8/38)	0.231	0.540 (0.193–1.511
Implantation rate	26.4 (48/182)	21.9 (47/215)	0.294	1.280 (0.807–2.032)
Ectopic pregnancy rate	1	0	/	/
Total number of live-born babies Singletons Twins	41 37 2	38 30 4	0.162	0.875 (0.722–1.061)
Gestation at delivery (completed weeks)	38.57 (37.57–39.57)	38.57 (37.14–39.14)	0.478	
Birth weight (g)	3,190 (2,915–3,375)	3,110 (2,501–3,328)	0.118	
Male babies	26/41 (65.4)	24/38 (63.1)	0.981	0.996 (0.712–1.394)

Data are presented as the number (percentage) or mean (data range).

CI, conﬁdence interval; FET, frozen–thawed embryo transfer; HA, hyaluronic acid.

### 3.3 Secondary outcome

The implantation rate, multiple pregnancy rate, and ectopic pregnancy rate were similar between the two groups (*p* > 0.05). Forty-one and 38 infants were born in the HA group and control group, respectively; among them, 37 were singletons and two were twins in the HA group, and 30 were singletons and four were twins in the control group (*p* = 0.162; relative risk 0.875; 95% CI 0.722–1.061). The gestational age at delivery, birth weight, and proportion of male infants was also similar between the two groups (**Table 3**). We also performed subgroup analyses by stratifying women into different types of embryos transferred (cleavage embryos versus blastocysts). The clinical pregnancy rates, live birth rates, miscarriage rates, implantation rates, and multiple pregnancy rates in these subgroup analyses were comparable between the HA and control groups (*p* > 0.05) (**Figures S1**, **S2**).

### 3.4 Logistic regression

The multivariate logistic regression model using the enter method by maternal age, BMI, previous transfer cycles, the number of previous embryos transferred, previous pregnancy, the duration of infertility, the cause of infertility, the antral follicle count, basal FSH levels, the insemination method, the duration of FSH stimulation, the total dosage of FSH, serum oestradiol/progesterone levels on the day of trigger, the number of oocytes collected, the methods of endometrial preparation, peak serum oestradiol levels in the FET cycle, the presence of top-quality embryos after thawing, the use of HA-enriched transfer medium, endometrial thickness, D3/D5 transfers, the number of embryos replaced and the number of embryos frozen. The results revealed that number of previous embryos transferred, endometrial thickness, the number of blastocysts transferred, and use of HA-enriched transfer medium were associated with the clinical pregnancy rate per transfer after adjusting for other confounding factors (**Table 4**). Only the number of blastocysts transferred, endometrial thickness, and the number of previous transfer cycles, and not the use of HA-enriched transfer medium, were associated with the live birth rate per transfer (**Table 5**).

**Table 4:** Logistic regression of clinical pregnancy rate in FET cycles.

Logistic regression analysis	β	*p*-Value	OR	95% CI
Maternal age at the time of IVF	−0.032	0.516	0.969	0.880–1.066
Duration of infertility	−0.065	0.395	0.937	0.806–1.089
Body mass index	−0.145	0.092	0.865	0.732–1.024
Insemination method	−0.726	0.061	0.484	0.226–1.035
Previous pregnancy	−0.446	0.224	0.640	0.312–1.314
No. of previous transfer cycles	−0.941	0.006	0.390	0.199–0.768
Number of previous embryos transferred	0.367	0.032	1.443	1.033–2.016
Cause of infertility		0.620		
Tubal			1	
Anovulatory Male Endometriosis Unexplained	0.034 −0.669 −0.444 0.958	0.963 0.279 0.516 0.401	1.035 0.512 1.559 2.606	0.240–4.460 0.153–1.719 0.409–5.945 0.279–24.376
Endometrial thickness	0.273	0.005	1.314	1.086–1.590
Blastocyst transfer	1.004	0.019	2.730	1.179–6.318
Presence of top-quality embryos after thawing	0.554	0.190	1.740	0.759–3.985
No. of embryos transferred	0.772	0.048	2.165	1.008–4.648
Stimulation regimen		0.214		
Long GnRH agonist			1	
GnRH antagonist	0.429	0.324	1.535	0.654–3.603
Mild stimulation	0.229	0.737	1.257	0.332–4.765
PPOS	−1.480	0.075	0.228	0.044–1.164
Short agonist	0.453	0.750	1.574	0.097–25.606
Endometrial preparation		0.076		
Natural cycle			1	
Hormonal cycle Mild stimulation	−0.794 −2.586	.125 .034	0.452 0.075	0.164–1.247 0.007–0.825
Use of HA-enriched transfer medium	0.816	0.027	2.260	1.097–4.659
Number of fresh embryo transfers performed	0.741	0.162	2.097	0.742–5.928
Duration of FSH stimulation	0.021	0.863	1.021	0.807–1.292
Total dosage of FSH	0.000	0.781	1.000	0.999–1.001
Number of oocytes collected	−0.028	0.636	0.972	0.972–1.092
Number of embryos frozen	0.048	0.613	1.049	0.872–1.261
AFC	0.007	0.872	1.007	0.924–1.097

Data are presented as the number (percentage) or mean ± SD.

FET, frozen–thawed embryo transfer; OR, odds ratio; HA, hyaluronic acid; IVF, *in vitro* fertilisation; FSH, follicle-stimulating hormone; GnRH, gonadotropin-releasing hormone; PPOS, progestin-primed ovarian stimulation; AFC, antral follicle count.

**Table 5:** Logistic regression of the live birth rate in FET cycles.

Logistic regression analysis	β	*p*-Value	OR	95% CI
Maternal age at the time of IVF	−0.050	0.314	0.952	0.864–1.048
Duration of infertility (years)	−0.043	0.573	0.958	0.824–1.113
Number of blastocysts transferred	1.012	.018	2.751	1.192–6.346
Presence of top-quality embryos after thawing	0.537	0.204	1.711	0.746–3.924
Endometrial preparation		0.138		
Natural cycle			1	
Hormonal cycle Mild stimulation	−0.704 −2.228	0.166 0.068	0.495 0.108	0.182–1.341 0.010–1.178
Use of HA-enriched transfer medium	0.547	0.134	1.728	0.845–3.533
Endometrial thickness	0.221	0.018	1.247	1.039–1.497
Cause of infertility		0.602		
Tubal			1	
Anovulatory Male Endometriosis Unexplained	−0.500 −0.737 0.588 0.006	0.529 0.244 0.379 0.996	0.606 0.479 1.801 1.007	0.128–2.877 0.139–1.653 0.486–6.673 0.085–11.985
Previous pregnancy	−0.405	0.267	0.667	0.326–1.364
Stimulation regimen		0.413		
Long GnRH agonist			1	
GnRH antagonist	0.264	0.537	1.303	0.563–3.016
Mild stimulation	3.016	0.797	1.189	0.317–4.463
PPOS	−1.278	0.120	0.279	0.056–1.397
Short agonist	0.509	0.720	1.664	0.103–26.806
BMI	−0.153	0.081	0.858	0.723–1.019
AFC	0.014	0.745	1.014	0.931–1.105
Duration of FSH stimulation	−0.014	0.907	0.986	0.781–1.246
Total dosage of FSH	0.000	0.897	1.000	0.999–1.001
Insemination method	−0.357	0.357	0.700	0.327–1.496
No. of previous transfer cycles	−0.772	0.024	0.462	0.236–0.905
Number of previous embryos transferred	0.295	0.086	1.343	0.959–1.881
Number of fresh embryo transfers performed	0.726	0.179	2.066	0.717–5.955
No. of embryos transferred	0.717	0.069	2.048	0.947–4.431
Number of oocytes collected	−0.018	0.765	0.983	0.876–1.103
Number of embryos frozen	0.018	0.845	1.018	0.849–1.221
Serum oestradiol level on the day of trigger	0.000	0.650	1.000	1.000–1.000
Serum progesterone level on the day of trigger	−0.012	0.978	0.988	0.438–2.229
Peak serum oestradiol level in FET cycle	0.001	0.152	1.001	1.000–1.001

Data are presented as the number (percentage) or mean ± SD.

FET, frozen–thawed embryo transfer; OR, odds ratio; HA, hyaluronic acid; IVF, *in vitro* fertilisation; FSH, follicle-stimulating hormone; GnRH, gonadotropin-releasing hormone; PPOS, progestin-primed ovarian stimulation; BMI, body mass index; AFC, antral follicle count.

## 4 Discussion

This retrospective study found an improvement in the clinical pregnancy rate when the HA-enriched transfer medium was used compared with the conventional transfer medium containing a low concentration of HA in patients with RIF undergoing FET cycles. In addition, the HA-enriched transfer medium showed positive treatment effects but no significant results in the live birth rate. The embryo implantation, clinical miscarriage, and multiple pregnancy rates were also similar between the two groups.

Our results are in agreement with those of previous studies (30), which showed that the addition of HA to the transfer medium in patients with RIF significantly increased the implantation and clinical pregnancy rates while not affecting the ectopic pregnancy and abortion rates. The mechanism of this beneficial effect might be that the addition of HA to the transfer medium increases its viscosity, which may enhance the embryo transfer process and reduce the possibility of the expulsion of embryos from the uterine cavity after transfer. Furthermore, HA increases cell–cell and cell–matrix adhesion and may improve embryo apposition and attachment (10). Considering the physical and chemical properties of HA, it may play a role in the embryo–endometrium interaction during the early phase of implantation, especially in patients with RIF (20). In contrast, a number of studies have found that the addition of HA to the transfer medium has no effect on pregnancy outcomes (31). Hambiliki et al. reported that although significantly higher positive hCG and implantation rates were seen after transfers with an HA-enriched medium, this did not result in a higher clinical pregnancy rate (32). It can be speculated that the addition of HA to the transfer medium may favour the attachment of early embryos that are intrinsically abnormal and that these embryos may implant but will be arrested in development later on.

In fact, the most important outcome measure that should be addressed is the live birth rate. However, in this study, we did not find any significant differences in the live birth rate between the use and non-use of HA-enriched transfer medium in FET cycles. This may be a result of the large proportion of pregnancies that fail to progress to birth after the use of HA, although the clinical pregnancy rate was found to be improved from 27.7% to 39.6% after the addition of HA in patients with RIF. In addition, the sample size of this study was too small to detect a difference of 9% in the live birth rate and might not have been able to detect a smaller difference, such as a difference of 5%–6% in the implantation rate. There might be a greater difference in the live birth rate when more patients are included.

The reasons why HA may only have beneficial effects for RIF patients remain unclear. However, as HA increases the pregnancy rate, we hypothesise that HA may increase the potential of embryos for implantation. Nakagawa et al. postulated that inadequate levels of HA might explain RIF in some patients and that HA can improve outcomes in this patient population (33). It is possible that in patients with RIF, the embryo secretion of HA is insufficient. HA is a known factor related to embryo implantation, and thus, the IR is improved in cases where there is a deficiency in HA secretion. Some researchers also reported that first-time implantation failure was not associated with a lack of HA secretion, and thus, a high HA concentration did not affect embryo development, which is a potential reason why an HA-enriched medium is not beneficial for patients with one implantation failure (34).

The cause of RIF can be attributed to two main factors, namely, dysfunction of the embryo and the endometrium (3). With the development of ART, it is less difficult to obtain high-quality embryos. Therefore, endometrial receptivity has become a key factor for the success of embryo transfer (35). Apart from embryo factors, uterine factors, including polyps, myomas, and adhesions, can also affect the implantation rate (36). Although our study excluded the above factors, some unknown factors, for example, embryo aneuploidy or immune factors, may affect embryo implantation. However, such RIF patients were not excluded in this study, which might have some impact on our results. Similar to other retrospective studies, patient selection bias was another limitation of this study; however, logistic regression analysis was carried out to control the bias possibly produced by imbalanced characteristics between the two groups. Nevertheless, this study might shed light on further research on RIF patient management. An RCT with a larger sample size is needed in the future to verify our findings.

## 5 Conclusion

Our findings supported the conclusion that HA can improve the clinical pregnancy rate in patients with RIF undergoing FET cycles, but the live birth rate was not significantly improved with the addition of HA to the traditional transfer medium. Future studies with more participants should be conducted to further confirm the beneficial effect of HA for RIF patients.

## Data availability statement 

The raw data supporting the conclusions of this article will be made available by the authors, without undue reservation.

## Ethics statement

Ethical approval (ethics number: KS22284) for the collection of human data was supported by the Ethics Committee of the Maternity and Infant Hospital. The requirement of written informed consent was waived due to the retrospective nature of the study, and patients’ data were used anonymously.

## Author contributions

KL and ZC were responsible for the conception and design of the study. QY, MZ, FH, RZ, XT, BH, and CZ were responsible for the acquisition of data. QY and ZC performed the data analysis and drafted the manuscript. KL and ZC revised and commented on the draft. All authors contributed to the article and approved the submitted version.
