# Supplementary figures and images for: Effect of hyaluronic acid-enriched transfer medium on frozen–thawed embryo transfer outcomes in RIF patients: a single-centre retrospective study
Source: Front Endocrinol (Lausanne). 2023 Jul 3;14:1170727. doi: 10.3389/fendo.2023.1170727 (PMC10350524; doi:10.3389/fendo.2023.1170727)

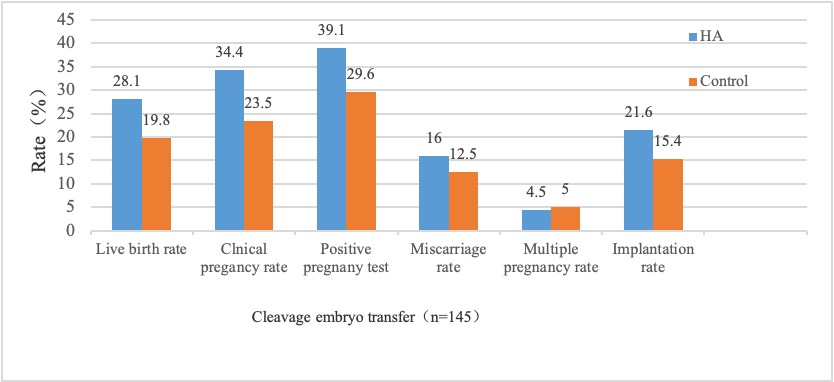

Supplement: Supplementary file 1 [file Image_1.jpeg]

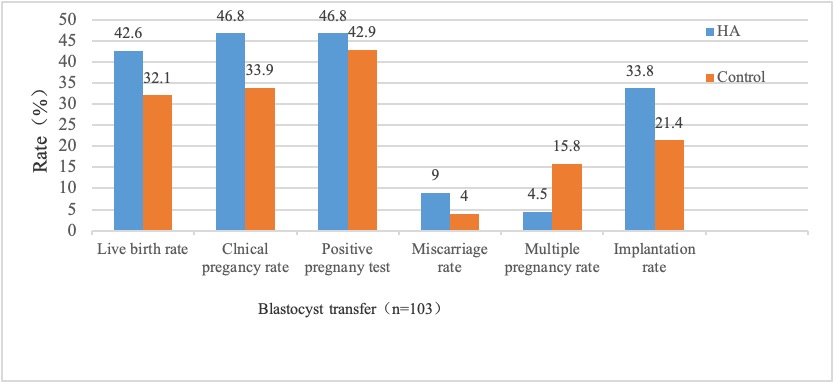

Supplement: Supplementary file 2 [file Image_2.jpeg]
